# Blood pressure control in hypertensive patients within Family Health Program versus at Primary Healthcare Units: analytical cross-sectional study

**DOI:** 10.1590/S1516-31802012000300003

**Published:** 2012-07-12

**Authors:** Telma Lima Martins, Álvaro Nagib Atallah, Edina Mariko Koga da Silva

**Affiliations:** I MSc. Assistant professor in the Discipline of Semiology and Clinical Medical, Faculdade de Medicina de Petrópolis (FMP), Petrópolis, Rio de Janeiro, Brazil.; II MD, PhD. Full professor and Head of the Discipline of Emergency Medicine and Evidence-Based Medicine, Universidade Federal de São Paulo - Escola Paulista de Medicina (Unifesp - EPM), São Paulo, Brazil.; III MD, PhD. Physician in the Discipline of Emergency Medicine and Evidence-Based Medicine, Universidade Federal de São Paulo - Escola Paulista de Medicina (Unifesp - EPM), São Paulo, Brazil.

**Keywords:** Hypertension, Health plan implementation, Public health, Population control, Health education, Hipertensão, Implementação de plano de saúde, Saúde pública, Controle da população, Educação em saúde

## Abstract

**CONTEXT AND OBJECTIVE:**

Hypertension is a public health problem due to its high prevalence and long-term cardiovascular complications. In Brazil in 2005, cardiovascular diseases were responsible for 28% of all deaths. Efforts are being made within primary care to achieve adequate hypertension control. The Family Health Program (FHP) has the aims of promoting quality of life and intervening in factors that put this at risk. The objective of this study was to evaluate the rate of blood pressure control among patients followed up at FHP units compared with those at primary healthcare units (PHUs).

**DESIGN AND SETTING:**

Analytical cross-sectional study in the municipality of Petrópolis, Rio de Janeiro, from January to December 2005.

**METHODS:**

Five hundred patients with a diagnosis of hypertension were included: 250 were being followed up at two FHP units and 250 at two PHUs. The diagnosis of hypertension was based on the Fourth Brazilian Hypertension Consensus, and the patients needed to have been under follow-up at the units for at least 12 months. Patients’ blood pressure was considered to be under control if it was less than 140/90 mmHg at the last consultation.

**RESULTS:**

Blood pressure was under control in 29.2% (n = 73) at FHP units and 39.23% (n = 98) at PHUs (odds ratio = 0.64; confidence interval = 0.44-0.93; P = 0.024).

**CONCLUSION:**

Blood pressure control was better among patients followed up at PHUs than among those followed up at FHP units.

## RESUMO

**CONTEXTO E OBJETIVO:**

A hipertensão arterial (HA) é um problema de saúde pública por sua elevada prevalência e complicações em longo prazo. No Brasil as doenças cardiovasculares foram responsáveis, em 2005, por 28% do total de óbitos em geral. Esforços vêm sendo implementados na atenção básica para o seu adequado controle. O Programa de Saúde da Família tem o objetivo de promover qualidade de vida assim como intervir nos fatores que a coloquem em risco. O objetivo deste estudo foi verificar a taxa de controle da HA em pacientes acompanhados nas Unidades de Saúde da Família (USF) comparados com pacientes acompanhados nas Unidades Básicas de Saúde (UBS).

**TIPO DE ESTUDO E LOCAL:**

Estudo transversal analítico, no município de Petrópolis, Rio de Janeiro, entre janeiro e dezembro de 2005.

**MÉTODOS:**

Foram incluídos 500 pacientes com diagnóstico de HA, sendo 250 em acompanhamento em duas USF e 250 em duas UBS. O diagnóstico de HA foi baseado no IV Consenso Brasileiro de Hipertensão e os pacientes precisavam estar em acompanhamento nas Unidades por no mínimo 12 meses. Foram considerados controlados os pacientes que apresentaram níveis pressóricos inferiores a 140/90 mmHg na última consulta.

**RESULTADOS:**

A taxa de controle da pressão arterial foi de 29,2% (n = 73) nas USF e de 39,23% (n = 98) nas UBS (*odds ratio* = 0,64; intervalo de confiança = 0,44-0,93), P = 0,024).

**CONCLUSÃO:**

Foi observado melhor controle da pressão arterial nos pacientes acompanhados nas UBS quando comparados aos pacientes acompanhados nas USF.

## INTRODUCTION

In the year 2000, it was estimated that hypertension would affect around 972 million adults worldwide: 333 million of them in economically developed regions and 639 million in developing regions. By 2025, a 60% increase in this total is expected, which would represent around 1.56 billion adults affected.^1^

According to data from the Brazilian Ministry of Health’s mortality information system (Sistema de Informação sobre Mortalidade, SIM) in 2005, cardiovascular diseases were responsible for around 28% of all deaths, i.e. 283,927 individuals. Among these, the main causes were acute myocardial infarction and stroke.^2^ In the State of Rio de Janeiro, out of the total number of deaths in 2005, 29% were due to cardiovascular causes.^2^

Hypertension is one of the most important and most prevalent risk factors for the development of atherosclerosis. It is the pathogenic basis for ischemic heart disease, cerebrovascular disease, kidney failure and peripheral vascular disease. It is chronic in nature and generally develops without symptoms over many years. Its high morbidity-mortality only comes to light 15 to 20 years after it starts. For all these reasons, it is a public health challenge worldwide. Therefore, all efforts towards early detection of this disease, with appropriate treatment and adherence to treatment, are justified. The aim behind such efforts is to control blood pressure levels and have a favorable impact on the cardiac, cerebrovascular, renal and peripheral vascular complications of this disease.^1^^,^^2^^,^^3^

Two recent studies have found a high prevalence of hypertension among the adult Brazilian population: 33.7% and 36.5%, taking high blood pressure to be levels of 140/90 mmHg and over.^4^^-^^5^ We were unable to find any national data referring to blood pressure control, but a cross-sectional population-based study was conducted among 918 adults over the age of 20 years in the State of Rio Grande do Sul in 1999-2000, taking hypertension to be blood pressure ^3^ 140/90 mmHg or to be represented by current use of antihypertensives. This study found that 49.2% did not know that they were hypertensive; 10.4% knew but were not following any treatment; 30.1% were following a course of treatment but did not present adequate control; and 10.4% were following a course of treatment with good control.^4^

Health programs and policies for controlling hypertension aim to diminish the complications, hospital admissions and deaths relating to hypertension. Furthermore, they aim to reduce the prevalence of hypertensive disease; increase the degree of knowledge among the population regarding the importance of controlling blood pressure; ensure access to primary healthcare services and medications for hypertensive individuals; and encourage community-based programs.

The Brazilian Family Health Program (FHP) began in 1994. Its aim is to promote healthcare for individuals in a holistic, integrated and continuous manner, through care provision for families and communities. It also aims to improve quality of life and focus on biopsychological, economic, cultural and social issues.^6^

The minimum team at FHP units is composed of a physician, a nurse, one or two nursing auxiliaries and four to six health agents, who work not only at the units but also within the community, through home visits.^6^ Thus, FHP units differ radically from Primary Healthcare Units (PHUs), where the team acts only in the unit and is composed of a general physician, a pediatrician and a gynecologist, along with a nursing auxiliary and a practice attendant.

A plan for redirecting care provision for hypertension and diabetes mellitus (HiperDia) was implemented by the Ministry of Health in 2002, within the Brazilian national health system (Sistema Único de Saúde, SUS), i.e. both in PHUs and in FHP units. This had the aim of providing technical support for professionals working within the primary care network, with regard to attendance not only for cases of hypertension, but also for diabetes mellitus, which is another public health problem.^7^

## OBJECTIVE

The objective of the present study was to evaluate the rate of controlled hypertension among hypertensive patients who were followed up at FHP units, comparing this with the rate of such control among patients followed up at traditional PHUs.

## METHODS

An analytical cross-sectional study was carried out in the city of Petrópolis, State of Rio de Janeiro, from January to December 2005. The research protocol was approved by the Research Ethics Committee of Universidade Federal de São Paulo - Escola Paulista de Medicina (Unifesp-EPM).

This study included 500 patients with hypertension as defined by the Fourth Brazilian Hypertension Consensus: 250 of them were being followed up at FHP units and 250 were being followed up at PHUs, in Petrópolis.

Public-sector healthcare establishments that had been in operation for more than 12 months were selected. The FHP units needed to have a complete team (one physician, one nurse, one to two nursing auxiliaries and four to six health agents). For the PHUs, the inclusion criterion was that they should have at least one general clinician. Two FHP units (Vila Saúde and Estrada da Saudade II) and two PHUs (Quitandinha and Dr. Thouzet) were randomly selected. The units were initially included by means of a draw. The draw was carried out using the following method: 1) the units were classified as either FHP units or PHUs; 2) the units were sequentially numbered; 3) each of these numbers was placed separately in a medium-brown opaque envelope without any identification. After the draw, the units were submitted to the inclusion and exclusion criteria of the study.

We took the criteria of 85% power and 5% alpha error to calculate our sample size and, thus, 222 patients in each group would be enough to demonstrate our hypothesis. An additional 12% were included to account for potential withdrawals and dropouts among the participants.

Each group was composed of 250 patients of both genders aged over 18 years with a diagnosis of primary hypertension, independent of any presence of comorbidities. All the patients selected had been undergoing follow-up for at least 12 months at the units.

The data collected from the medical files included: blood pressure at the first consultation; blood pressure at the last consultation; medication prescribed at the penultimate consultation; number of medical consultations over the past year; number of nursing consultations over the past year; and number of participations in group activities over the past year. Medical team member experience and qualifications were verified by directly asking each member for this information.

The following individuals were excluded: patients under 18 years of age, patients who had been followed up for less than 12 months, patients who lived outside of the city, patients with a diagnosis of secondary hypertension, pregnant patients and patients whose pressure levels had not been recorded.

The outcome evaluated was blood pressure control. Patients presenting at least one record of blood pressure less than 140/90 mmHg in their medical files from the last consultation, after a minimum of 12 months of follow-up, were deemed to present controlled pressure, in accordance with the advice contained in the Fourth Brazilian Hypertension Consensus.^8^

The blood pressure measurement equipment used in the units was of aneroid type (Certified or Missouri models), and the units affirmed that these devices were calibrated every six months. NAWA stethoscopes were used.

The statistical calculations were performed using the Vassar Stats Statistical Tables Calculator.^9^

## RESULTS

There was no statistical difference according to sex or age in the two study groups (Table 1).

**Table 1.** Characteristics of the populations studied at the Family Health Program (FHP) units and the primary healthcare units (PHUs). City of Petrópolis, January to December, 2005

Selected characteristics	FHP units (n = 250)		PHUs (n = 250)		P
	n	%	n	%	
Gender					
Male	78	31.2	80	32	0.923^*^
Female	172	68.8	170	68	
Age					
Mean age: men	62.0	(± 12.2)	62.0	(± 10.6)	0.263^†^
Mean age: women	58.0	(± 14.3)	61.5	(± 10.5)	

^*^Pearson chi-square test; ^†^Student’s t test for difference in means between pairs of groups: FHP units versus PHUs.

Blood pressure control among hypertensive patients at PHUs was higher than at FHP units (P = 0.024), and it was better among the men at PHUs (P = 0.007) (Table 2).

**Table 2.** Proportion of patients with blood pressure under control at the Family Health Program (FHP) units and the primary healthcare units (PHUs). City of Petrópolis, January to December, 2005

Selected characteristics	FHP units (n = 250)		PHUs (n = 250)		P
	n	%	n	%	
Blood pressure control					
Present	73	29.2	98	39.2	0.024
Absent	177	70.8	152	60.8	
Control among males					
Present	15	19.2	32	40.0	0.007
Absent	63	80.7	48	60.0	
Control among females					
Present	58	33.7	66	38.8	0.385
Absent	114	66.2	104	61.1	

Pearson chi-square test.

Regarding medical consultations, we observed that there were more consultations among patients followed up at FHP units (P = 0.011). Only the patients followed up at FHP units had nursing consultations, group activities or home visits (Table 3).

**Table 3.** Mean numbers of medical consultations, nursing consultations, participations in group activities and home visits among patients with blood pressure under control at the Family Health Program (FHP) units and the primary healthcare units (PHUs). City of Petrópolis, January to December, 2005

Selected characteristics	FHP units (n = 73)		PHUs (n = 98)		P
	mean	(SD)	mean	(SD)	
Medical consultations	4.1	(? 2.7)	3.0	(? 1.4)	0.011
Nursing consultations	1.6	(? 1.9)	Not applicable		
Participation in group activities	0.4	(? 1.2)	Not applicable		
Home visits	0.3	(? 0.8)	Not applicable		

Student’s t test for difference in means between pairs of groups: FHP units versus PHUs.

There were no statistical differences in relation to monotherapy, use of two drugs or use of more than two drugs, among the groups followed up at PHUs and FHP units (Table 4).

**Table 4.** Proportions of use of monotherapy or combinations of drugs among patients with blood pressure under control at the Family Health Program (FHP) units and the primary healthcare units (PHUs). City of Petrópolis, January to December, 2005

Selected characteristics	FHP units (n = 73)		PHUs (n = 98)		P
	n	%	n	%	
Monotherapy	34	46.7	40	40.0	0.945
Two drugs	32	43.8	53	54.0	
> two drugs	7	9.5	5	5.1	

Pearson chi-square test.

There was also no statistical difference regarding classes of antihypertensive drugs, either in monotherapy or in associations (Table 5).

**Table 5.** Proportions of drug classes used for monotherapy and combinations among patients with blood pressure under control at the Family Health Program (FHP) units and the Primary Healthcare Units (PHUs). City of Petrópolis, January to December, 2005

Selected characteristics	FHP units (n = 34)		PHUs (n = 40)		P
	n	%	n	%	
Monotherapy					
ACEI	19	55.8	29	72.5	0.916
Diuretic	9	26.4	3	7.5	
Calcium channel blocker	3	8.8	3	7.5	
Beta blocker	2	5.8	4	10.0	
Central blocker	1	2.9	1	2.5	
	FHP units (n = 32)		PHUs (n = 53)		P
	n	%	n	%	
Two drugs					
ACEI with diuretic	21	65.6	18	33.9	0.834
ACEI with calcium blocker	2	6.2	6	11.3	
ACEI with central blocker	0	00	5	9.4	
ACEI with beta blocker	1	3.1	3	5.6	
ACEI with vasodilator	1	3.1	0	0	
ACEI with others	1	3.1	0	0	
Diuretic with beta blocker	2	6.2	3	5.6	
Diuretic with calcium blocker	4	12.5	5	9.4	
Diuretic with central blocker	0	0	9	16.9	
Other associations	0	0	4	7.5	

Pearson chi-square test. ACEI = angiotensin-converting enzyme inhibitor.

## DISCUSSION

We observed in our study that the proportion of the patients with blood pressure that was under control at the last consultation at the FHP units was 29.2%, while at the PHUs, this rate was 39.2%. Although the observed percentage control was unsatisfactory, it was similar to what has been described in the literature. American data from National Health and Nutrition Examination Survey (NHANES) 2003-2004 showed controlled blood pressure in 36.8%,^9^ while Brazilian data from a study in the State of Rio Grande do Sul showed a control rate of 10.4%.^4^

We observed that the blood pressure control was better among the men studied at PHUs. However, we were unable to explain this finding, taking into account the size of the sample.

The attendance model proposed for the FHP aims towards health promotion through team actions relating to quality of life, with interventions applied to factors that place this quality of life at risk. This is to be achieved through knowing the clientele better, not only at the units but also in their homes, and through detecting these people’s real needs and encouraging them to recognize that their health and quality of life are citizens’ rights. With this model in mind, it was expected that when the HiperDia program was implemented within SUS, the FHP units would be more effective in controlling blood pressure, compared with the traditional model of the PHUs. The teams at PHUs are not multidisciplinary and they act only in the PHUs: there are no consultations at patients’ homes and no active searches for missing patients are conducted. However, what we found was that the blood pressure control at the FHP units was inferior to the control achieved at the traditional PHUs.

Our study compared populations that were very similar, formed by individuals who sought primary healthcare through SUS and who therefore were of comparable socioeconomic level. Furthermore, the groups were similar in terms of gender and age distribution. Access to medications at the two types of unit (FHP units and PHUs) is identical, since both types form part of the Ministry of Health’s HiperDia program. The medications provided are supplied by the city health authorities and the state government. The HiperDia manual, containing guidance relating to diagnosing and managing high blood pressure, was available at all the units evaluated.

With regard to the medical professionals working in the two types of unit, we observed that they presented different characteristics, such as the length of time since graduation and the different specialties represented. Differences in specialties lead to the hypothesis that the results encountered might have been influenced by this factor, but in this respect, not only the physicians’ original training but also their continuing training would have to be taken into account. Davis and Taylor-Vaisey^10^ suggested that continuing education among physicians leads to better performance in relation to treatment for cardiovascular disease and in relation to dealing with its risk factors. Schneider et al.^11^ showed through a questionnaire answered by emergency department physicians and general clinicians that only 36% correctly knew the levels that define high blood pressure. The latter study took high blood pressure to be > 140/90 mmHg.

Data from evaluations in 167 countries published by the World Health Organization (WHO) in 2003^12^ showed that general physicians were unaware of national consensuses on hypertension in 61% of the countries and that in 45% of them professionals were not trained to manage hypertension. Data from the Brazilian Ministry of Health^13^ published in 2004 showed that between 2001 and 2002, the introductory training provided by the ministry, which ought to be given before or immediately after setting up the teams at the FHP units, reached averages of 61.9% of the physicians and 69.4% of the nurses working within the FHP nationwide. Specific training for these teams in relation to managing hypertension reached averages of only 42.4% of the physicians and 44.5% of the nurses, nationally. In the State of Rio de Janeiro, these averages went up to 50.5% and 51.6%, respectively.^13^

In the city of Petrópolis, the introductory course was given at the time of implementing the program in 1997, but the Ministry of Health’s specialization course on Family Medicine was only given in 2002. Continuing healthcare education has been provided over this period, with material from the Ministry of Health and delivery by professionals from within the public healthcare system.

In the 36 FHP units set up in the city of Petrópolis up to 2005, the majority of the physicians working in the teams had not participated in the specialization course on Family Medicine that the Ministry of Health provided in 2002. Most of the physicians working in the nine PHUs of the municipality had been trained in internal medicine.

We observed that the larger number of consultations that took place at the FHP units, in relation to the number of consultations at PHUs, was not reflected in better control over hypertension. We suspect that both the quality of the consultation and the physicians’ training were factors that may have influenced the results.

Haynes demonstrated that despite the known need for adherence to treatment in order to control high blood pressure, there was great difficulty in achieving this. Several models have been tested with a view to improving the adherence to treatment for chronic diseases. Complex strategies combining different approaches have been most successful and have increased adherence by 23% to 50%.^14^^,^^15^^,^^16^ Such strategies have included: motivational planning by the healthcare team, reminders for patients by means of telephone calls or leaflets, self-determination by patients, self-measurement of pressure, monthly visits, counseling, social or family support, and time availability and dedication of a trained team.

Another important point regarding adherence to treatment for these diseases relates to the drugs used and their prescription. A meta-analysis conducted by Schroeder,^17^ in which drugs administered once or twice a day were tested, showed a single study in which a decrease of 6 mmHg in systolic pressure, with important repercussions on diastolic pressure, was found with the use of drugs taken once a day.

Data from the Primary Care Department^18^ have shown that the drugs most used within HiperDia are ACE (angiotensin-converting enzyme) inhibitors, diuretics and beta blockers. In our study, the type of monotherapy most used was ACE inhibitors and the combination most used was ACE inhibitors with diuretics. However, the monotherapy did not show better blood pressure control, considering that the ACE inhibitor used was captopril, which has to be taken as at least three doses per day.

Our study presents certain limitations, given that the data were extracted from the medical files. Moreover, although both types of unit took their guidance from the HiperDia program, both for measuring blood pressure and for diagnosing hypertension and treating it, the blood pressure measurements were performed by different people and we cannot be absolutely sure that the diagnostic criteria and case management were followed equally in the two groups.

## CONCLUSION

The rate of blood pressure control among patients in FHP units in the city of Petrópolis was 29.2% and the rate of control in the PHUs was significantly higher (39.2%).

The results show that the level of hypertension control in both types of unit is still unsatisfactory. New studies are needed in order to identify the possible obstacles that may be influencing these results.
